# LINC00494 Promotes Ovarian Cancer Development and Progression by Modulating NFκB1 and FBXO32

**DOI:** 10.3389/fonc.2020.541410

**Published:** 2021-01-28

**Authors:** Yang Shu, He Zhang, Jinqiu Li, Yanhong Shan

**Affiliations:** ^1^Department of Obstetrics and Gynecology, The First Hospital of Jilin University, Changchun, China; ^2^Department of Gynecology, The First Hospital of Jilin University, Changchun, China; ^3^Department of Otolaryngology—Head and Neck Surgery, The Second Hospital of Jilin University, Changchun, China; ^4^Department of Obstetrics, The First Hospital of Jilin University, Changchun, China

**Keywords:** ovarian cancer, long non-coding RNA, LINC00494, NFκB1, F-box protein 32

## Abstract

**Background:**

Ovarian cancer represents one of the most frequent gynecological cancers and is significant cause of death for women around the world. Long non-coding RNAs (lncRNAs) are recognized as critical governors of gene expression during carcinogenesis, but their effects on the occurrence and development of ovarian cancer require further investigation. In this report, we characterized LINC00494 as a novel oncogenic lncRNA in ovarian cancer.

**Methods:**

Bioinformatics analysis predicted potential interactions among LINC00494, NFκB1, and FBXO32 in ovarian cancer, which were tested by dual-luciferase reporter assay, RNA pull-down, RIP, and ChIP assay. Cancer cells were transfected with relevant treated plasmids, followed by scratch and Transwell assays. The treated cells were injected into nude mice to establish a xenograft model for testing effects of LINC00494 and its target gene *in vivo*.

**Results:**

LINC00494 and NFκB1 were highly expressed whereas FBXO32 had low expression in ovarian cancer cells and tissues. LINC00494 was found to bind NFκB1 and increase its activity, while NFκB1 was enriched at the FBXO32 promoter region, where it acted to reduce FBXO32 transcription. Overexpression of LINC00494 elevated NFκB1 expression and enhanced cell migration, invasion and tumorigenesis, but additional overexpression of FBXO32 interfered with the tumorgenicity of ovarian cancer cells *in vitro* and *in vivo*.

**Conclusion:**

Our work demonstrated that LINC00494 promoted ovarian cancer progression by modulating FBXO32 *via* binding with the transcription factor NFκB1. These results provided new insight into the mechanism of ovarian cancer pathogenesis and suggested new therapeutic targets.

## Introduction

Ovarian cancer is one of the most frequently occurring gynecological cancers, and brings high mortality and morbidity to women the world over (1). Ninety percent of ovarian cancers are thought to arise from ovarian surface epithelium (OSE) cells (2). Most women diagnosed with ovarian cancer have already progressed to stage III (51%) or IV (29%) at diagnosis, and have a high rate of relapse and metastasis into the abdominal cavity (3). Ovarian cancer is a heterogenous malignancy that can be classified into at least five distinct histological subtypes that possess various recognized risk factors and unique genomic characteristics (4). The effectiveness of population-based screening for ovarian cancer has been disappointing, which calls for new approaches that leverage molecular genomics for early diagnosis and timely treatment of ovarian cancer (5).

To date, a very diverse group of long non-coding RNAs (lncRNAs) have emerged critical regulators of gene expression and pathway activation in human cancers, including ovarian cancer (6). The lncRNAs, which consist of at least 200 nucleotides, engage in a broad spectrum of genetic regulation, such as epigenetic, transcriptional, or post-transcriptional regulation, thus positioning them to facilitate tumor initiation and enhance metastatic potential (7). Ovarian cancer samples in the GSE38666 dataset deposited in the Gene Expression Omnibus (GEO) showed differential expression of LINC00494, which motivated us to characterize further its functional role in the pathogenesis of ovarian cancer. We predicted through the LncMAP database that NFκB1 was a putative transcriptional factor regulated by LINC00494, and that FBXO32 was the downstream target gene of NFκB1. The NF-κB family of transcription factors encompass five subunits, RelA (p65), c-Rel, RelB, NF-κB1 (p50 and its precursor p105), and NF-κB2 (p52 and its precursor p100), which participate in the formation of distinct homodimeric and heterodimeric complexes in mammalian cells (8). NF-κB1 was highly expressed in the context of ovarian cancer, and the extent of its overexpression at diagnosis predicts outcome (9, 10). FBXO32 (F-box protein 32) is a novel TGF-beta/SMAD4 target gene, which is recently recognized to be highly upregulated in human normal ovarian surface epithelium, where it functions as a tumor suppressor (11). From previous evidence, promoter hypermethylation-mediated loss of FBXO32 expression was linked with poor patient survival in human ovarian cancer (12). Therefore, we performed this study to define better the regulatory role of the LINC00494/NFκB1/FBXO32 axis in the development of ovarian cancer.

In the present study, we showed that LINC00494 expression was significantly upregulated in ovarian cancer, and its knockdown inhibited ovarian cancer cell migration, invasion and tumorigenesis *in vivo*.

## Materials and Methods

### RNA Interference and Overexpression

Normal ovarian epithelial cells IOSE80 and human ovarian cancer cell lines Caov-3, A2780, SKOV3, and CoC1 (COBIOER, Nanjing, China) were resuspended in calf serum-free RPMI1640 (PM150110, Procell Life Science & Technology, Wuhan, China). The cells were centrifuged at 1000 rpm for five min and incubated in cell medium with 15% fetal bovine serum (FBS) at 5% CO_2_ and room temperature. Then the Caov-3 cells and SKOV3 cells at logarithmic phase were seeded onto 6-well plates. When confluence reached 30%~60%, cells were transfected with short interfering RNA (si)-LINC00494, overexpression (oe)-LINC00494, oe-NFκB1, si-NFκB1, and oe-FBXO32 (Shanghai GenePharma Co, Ltd, Shanghai, China) using Lipofectamine 2000 reagents (#11669-027, Invitrogen, USA).

### Real-Time qPCR

RNA extractions from SKOV3 and Caov-3 cells were obtained using Trizol reagents (#15596-018, Invitrogen), and cDNA was generated using a commercially available kit (Thermo Scientific, USA) as per the manufacturer’s protocol. qRT-PCR was performed using SYBR^®^ Premix Ex TaqTM II (Perfect Real Time) kit (DRR081, Takara, Japan) and the reaction system kit (KR011A1, Beijing Puyihua Science and Technology, China) along with the Bio-Rad iQ5 PCR instrument (Bio-Rad, USA). Data were normalized to fold-change of the housekeeping gene GADPH, and determined using the delta-delta comparative threshold cycle (ΔΔCt) method. Primers (**Table 1**) were synthetized by Takara (Japan).

**Table 1:** Primer sequences used for qRT-PCR analysis.

RNA	Primer sequences (5’-3’)
LINC00494	F: 5’-TCCAAATCCCCAACCTGAGC-3’
	R: 5’-CTGGCCTTGCTCTAAGCACT-3’
NFκB1	F: 5’-CTGCTGACAATTTCCCACAC-3’
	R: 5’-GCTCTCTGAGCACCTTTGGA-3’
FBXO32	F: 5’-ATTGGTTAGTGATAGTTAAGGGT-3’
	R: 5’-GGGATAACGGTGTTTTGG-3’
GAPDH	F: 5’-ATGGAGAAGGCTGGGGCTC-3’
	R: 5’-AAGTTGTCATGGATGACCTTG-3’

F, forward; R, reverse.

### Immunoblotting

SKOV3 and Caov-3 cells were lysed in ice-cold radioimmunoprecipitation (RIPA) buffer supplemented with 1 mM freshly prepared phenylmethylsulfonyl fluoride. Equal amounts of protein samples (30 μg) in each well were run on 10% SDS-PAGE and then wet-transferred to the polyvinylidene fluoride (PVDF) membrane (P2438, Sigma-Aldrich, USA). Immunoblots were probed with anti-NFκB1 antibody (ab32360, Abcam, UK), anti-FBXO32 antibody (ab168372, 1:2000, Abcam), anti-GAPDH antibody (ab181602, Abcam), and reprobed with horseradish peroxidase-labeled IgG. GAPDH was used as a loading control for normalization. Next, immunoblots were visualized using the enhanced chemiluminescence (ECL) method (BB-3501, Ameshame, UK) as per the manufacturer’s protocol. The experiments were repeated three times.

### Luciferase Assays

The lncMAP website (http://bio-bigdata.hrbmu.edu.cn/LncMAP/lncrna_result.jsp) demonstrated that LINC00494 could regulate the expression of FBXO32 through the transcription factor NFκB1. The FBXO32 sequence was obtained from Gen Bank (National Center for Biotechnology Information database, Bethesda, Maryland, USA). Based on the prediction, we established the 3’UTR sequence of the FBXO32 promoter that contained NFκB1 binding sites. Then mutant FBXO32 3’UTR sequence (FBXO32-MUT) and wild type FBXO32 3’UTR sequence containing NFκB1 binding (FBXO32-WT) were cloned into luciferase reporter vectors (GUR100014-P-2, RIBOBIO, Guangzhou, China). Then, the custom-designed reporter vectors were co-transfected with plasmids expressing oe-NFκB1 and siNFκB1 into SKOV3 cells or Caov-3 cells. Firefly luciferase activity (FLUC) and Renilla luciferase activity Rellia (RLUC) were detected using Luminometer (TD20/20: Turner Designs, Sunnyvale, CA, USA). The ratio of RLUC/FLUC was considered as the relative fluorescence intensity.

### Chromatin Immunoprecipitation Assays

Enrichment of NFκB1 in the FBXO32 promoter region was evaluated by ChIP assays following the instructions provided by the kit’s manufacturer (Milliporeg, USA). In brief, SKOV3 and Caov-3 cells were fixed with 10% formaldehyde for ten min to generate DNA-protein cross-links. Cell lysates were then sonicated to generate chromatin fragments. The chromatin fragments were divided into three parts, of which two were immunoprecipitated either with an antibody against NFκB1 (ab32360, Abcam) or normal mouse IgG at 4°C overnight. The DNA-protein complexes were precipitated by using Protein Agarose/Sepharose. The immunoprecipitation was de-crosslinked, and the DNA samples were extracted by a standard phenol/chloroform procedure and analyzed by real-time qPCR analysis. The experiment was performed in triplicate.

### RNA Immunoprecipitation Assays

LINC00494 binding with NFκB1 was evaluated using RIP kit (Millipore, USA) according to the manufacturer’s instructions. Cells were lysed with RIPA lysis (P0013B, Beyotime Biotechnology Co., Ltd., Shanghai, China) and centrifuged (14,000 × g, 10 min). Cell extracts were co-precipitated with antibody NFκB1 (ab32360, Abcam) or rabbit-anti human IgG (1:100, ab109489, Abcam), and digested with trypsin K, followed by RNA extraction for real-time qPCR analysis to detect LINC00494. The experiment was performed in triplicate.

### RNA Pull-Down

LINC00494 binding with NFκB1 was evaluated by RIP assays. In brief, SKOV3 cells were treated with either biotin-labeled WT-bio-LINC00494 or MUT-bio-LINC00494 probes (50 nM) for 48 h, and then lysed and centrifuged (14,000 × g, 10 min). Cell extracts were immunoprecipitated with M-280 streptavidin beads (S3762, Sigma-Aldrich) pre-coated with RNase-free bovine serum albumin (BSA) and yeast tRNA (TRNABAK-RO, Sigma-Aldrich). Immunoprecipitated protein and total protein from the whole cell lysates (input controls) were extracted for immunoblotting analysis of NFκB1.

### Scratch Test

SKOV3 and Caov-3 cells maintained with serum-free medium were seeded into the 6-well plate with 1 × 10^5^ cells/well. A thin scratch (10 µl) was created along the center of each well with a sterile pipette tip (the width of each scratch was nearly the same). To evaluate wound closure, six fields were selected, and the cells were photographed at 0 and 24 h after incubation with serum-free medium. The cells in the wound area were counted and analyzed by counting software.

### Transwell Invasion Assays

SKOV3 and Caov-3 cells were prepared in a suspension of 1 × 10^5^ cells/ml using the serum-free DMEM and then added into the upper part of Transwell chambers coated with 30 μl Matrigel (40111ES08, YeaSen Biotechnology, Shanghai, China) pre-diluted (1:3) with serum-free DMEM. After 24-h incubation at 37°C, the cells that transferred to the lower chamber containing 0.5 ml 10% FBS-supplemented DMEM were fixed in 95% ethanol and stained with 0.1% crystal violet staining, and counted in six microscopic fields per well using an inverted microscope (Olympus, Tokyo, Japan).

### Mouse Xenograft Experiments

A total of 24 BALB/c mice (sex in half, aged 4-6 weeks; weighing 18 to 22 g, J004, Nanjing, Junke Biological Co., Ltd, Jiangsu, China) were kept under specific pathogen-free conditions. SKOV3 cells were transfected with oe-LINC00494 or si-LINC00494. Besides, SKOV3 cells not transfected with any vector were used as control (blank group). Then the treated cells were subcutaneously injected into the mice (n = 8, each group; male:female = 1:1). The growth of the ovarian cancer xenografts was monitored for six weeks after implantation, whereupon the mice were euthanized by cervical dislocation, with tumor bodies excised for examination. Animal studies were performed with the approval from the Institutional Animal Care and Use Committee of the First Hospital of Jilin University.

### Bioinformatics Analysis

Differentially expressed miRNAs (DEMs) related to ovarian cancer were screened using the GSE38666 miRNA expression profile downloaded from the GEO database (13). Epithelial tissue samples from GSE38666 consisted of 12 normal samples and 18 cancer samples. The limma package was employed for differential expression analysis with threshold set as |logFC| > 2 and p-value < 0.05. Expression of the differentially expressed genes was displayed in a heat map generated using the pheatmap package. Additionally, LINC00494 expression was detected in ovarian cancer samples and normal samples from TCGA and GTEx, using the GEPIA2 dataset (http://gepia2.cancer-pku.cn/#about) (14).

### Statistical Analysis

All statistical analyses were representative of three independent experiments (each in triplicate) and performed with SPSS 21.0 software (IBM, Armonk, NY, USA), with *p* < 0.05 as a level of statistical significance. For statistical comparisons, independent-sample t-test, a one-way analysis of variance (ANOVA) with Tukey’s *post hoc* test, and repeated measures ANOVA with Bonferroni *post hoc* test were performed as required.

## Results

### Upregulated LINC00494 and NFκB1 and Downregulated FBXO32 in Ovarian Cancer

**Figure 1:**
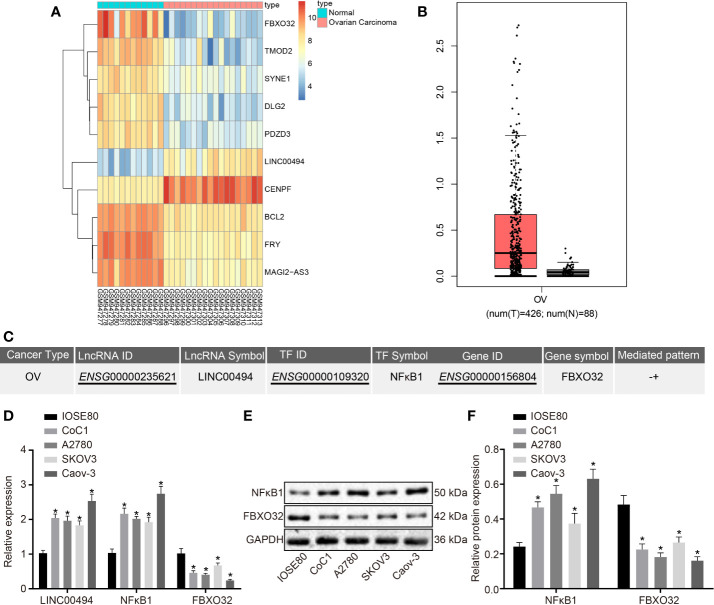
LINC00494 and NFκB1 were highly expressed and FBXO32 was lowly expressed in ovarian cancer. **(A)** Heatmaps of differentially expressed mRNA between ovarian cancer and normal ovarian tissue samples in the GSE38666. **(B)** Expression box of LINC00494 in the GEPIA database (http://gepia.cancer-pku.cn/). **(C)** A putative LINC00494/NFκB1/FBXO32 signaling pathway in the LncMAP database (http://bio-bigdata.hrbmu.edu.cn/LncMAP/lncrna_result.jsp). **(D)** The expression of LINC00494, NFκB1 and FBXO32 was compared between ovarian cancer cell lines and IOSE80 cells by real-time qPCR. **(E)** Immunoblots of NFκB1 and FBXO32 between ovarian cancer cell lines and IOSE80 cells, normalized to GAPDH. **(F)** Quantification of NFκB1 and FBXO32 between ovarian cancer cell lines and IOSE80 cells. * indicates *p* < 0.05 compared with IOSE80 cells by Tukey’s test-corrected one-way ANOVA.

We first downloaded an ovarian cancer-related microarray dataset (accession numbers: GSE38666) deposited in the GEO and performed differential expression analysis between ovarian cancer and normal ovarian tissue samples. The differentially analysis indicated high expression of LINC00494 and low expression of FBXO32 in ovarian cancer, which was entirely consistent with corresponding findings in clinical cancer tissues and normal tissues (**Figure 1A**). The GEPIA2 database analysis (http://gepia2.cancer-pku.cn/#index) showed that the expression of LINC00494 in ovarian cancer was higher levels relative to that in paracancerous tissues (**Figure 1B**). We next performed a computer-based prediction in the LncMAP database, which indicated NFκB1 as a putative transcriptional factor regulated by LINC00494, and that FBXO32 was the downstream target gene of NFκB1 (**Figure 1C**).

We performed real-time qPCR and immunoblotting analysis to determine the expression levels of LINC00494, NFκB1, and FBXO32 in several ovarian cancer cell lines (Caov-3, A2780, SKOV3, and CoC1) and a normal ovarian epithelial cell line, IOSE80. Relative to IOSE80, all cancer cell lines exhibited upregulated expression levels of LINC00494 and NFκB1 as well as downregulated expression level of FBXO32 (**Figures 1D–F**). Among the four ovarian cancer cell lines, SKOV3 cells exhibited the lowest expression level of LINC00494 and CaOV3 cells exhibited the highest expression. Therefore, Caov-3 and SKOV3 cells were chosen for further experiments.

### LINC00494 Facilitated Ovarian Cancer Cell Migration and Invasion

**Figure 2:**
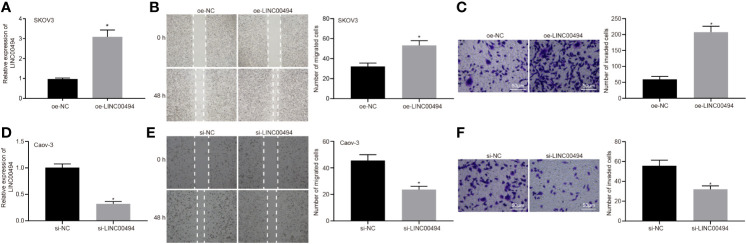
LINC00494 was capable of inducing SKOV3 and Caov-3 cell migration and invasion. **(A)** Verification of LINC00494-overexpressed SKOV3 cells by real-time qPCR. **(B)** LINC00494-overexpressed SKOV3 cell migration was measured by scratch healing test. **(C)** Representative view (× 200) and statistics of LINC00494-overexpressed SKOV3 cells invading from Matrigel-coated upper transwell chambers into lower ones. **(D)** Verification of LINC00494 knockdown Caov-3 cells by real-time qPCR. **(E)** LINC00494 knockdown Caov-3 cell migration was measured by scratch healing test. **(F)** Representative view (× 200) and statistics of LINC00494 knockdown Caov-3 cells invading from Matrigel-coated upper transwell chambers into lower ones. * indicates *p* < 0.05 compared with empty vectors **(A–C)** or with scramble siRNA **(D–F)** by unpaired t-test or Tukey’s *post hoc* correction.

Following the identification of high LINC00494 expression in ovarian cancer, we characterized the functional role of LINC00494 in ovarian cancer. SKOV3 cells were transfected with an expression vector containing oe-LINC00494. Real-time qPCR demonstrated that oe-LINC00494 effectively elevated LINC00494 expression in ovarian cancer cells (**Figure 2A**). The results of scratch and transwell invasion assays showed that overexpression of LINC00494 facilitated SKOV3 cell migration and invasion (**Figures 2B, C**). Likewise, LINC00494-specific siRNA was designed and delivered into Caov-3 cells for construction of LINC00494 knockdown ovarian cancer cells (**Figure 2D**). The results of scratch and transwell invasion assays showed that siRNA knockdown LINC00494 retarded Caov-3 cell migration and invasion (**Figures 2E, F**). Taken altogether, overexpression of LINC00494 could promote SKOV3 cell migration and invasion, while downregulation of LINC00494 could inhibit Caov-3 cell migration and invasion.

### LINC00494 Could Bind With NFκB1 and Increase Its Activity in Ovarian Cancer Cells

**Figure 3:**
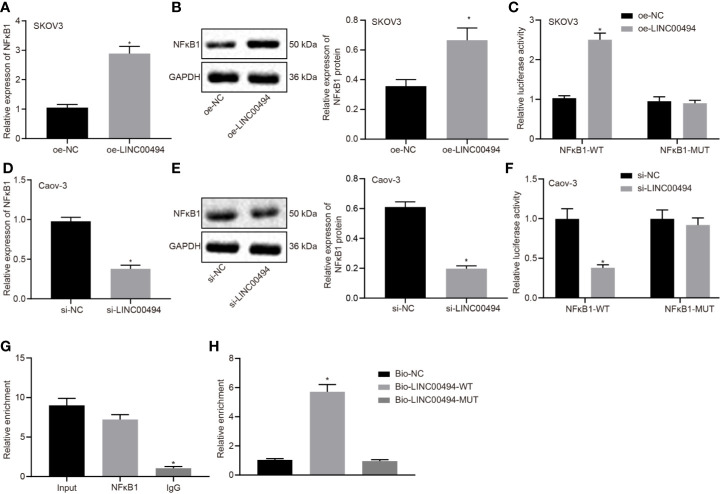
Interaction between LINC00494 and NFκB1 protein in ovarian cancer cells. **(A)** Real-time qPCR of NFκB1 expression in oe-LINC00494-treated SKOV3 cells. **(B)** Immunoblots of NFκB1 expression in oe-LINC00494-treated SKOV3 cells and corresponding quantification. **(C)** The luciferase luminescence at the promoter of the reporter gene containing NFκB1-WT and NFκB1-MUT in oe-LINC00494-treated SKOV3 cells. **(D)** Real-time qPCR of NFκB1 expression in si-LINC00494-treated Caov-3 cells. **(E)** Immunoblots and quantification of NFκB1 in si-LINC00494-treated Caov-3 cells. **(F)** The luciferase luminescence at the promoter of the reporter gene containing NFκB1-WT and NFκB1-MUT in si-LINC00494-treated Caov-3 cells. **(G)** LINC00494 was more enriched at the NFκB1 promoter region in the immunoprecipitation using NFκB1 antibody relative to IgG. **(H)** RNA pull-down assay of NFκB1 binding to LINC00494 probes. * indicates *p* < 0.05 compared with empty vectors **(A–C)**, scramble siRNA **(D–F)**, normal IgG **(G)**, or biotin-labeled NC **(H)** by unpaired t-test or Tukey’s test-corrected one-way ANOVA.

In the next experiments, we further investigated the molecular mechanisms behind LINC00494’s involvement in ovarian cancer. Following treatment of oe-LINC00494, SKOV3 cells showed an elevated expression of NFκB1 as determined by real-time qPCR and immunoblotting analysis (**Figures 3A, B**). Following LINC00494 overexpression, we observed enhanced luciferase activity at the promoter of the reporter gene containing NFκB1-WT in SKOV3 cells (**Figure 3C**). According to real-time qPCR and immunoblotting analysis, Caov-3 cells treated with si-LINC00494 exhibited reduced expression of NFκB1 and LINC00494 (**Figures 3D, E**). Treatment with si-LINC00494 diminished the luciferase activity of NFκB1-WT in Caov-3 cells, but no such effect was found in NFκB1-MUT (**Figure 3F**). Furthermore, RIP assays demonstrated more LINC00494 was enriched at the NFκB1 promoter region in the immunoprecipitation obtained with NFκB1 antibody relative to the IgG control (**Figure 3G**). As shown in **Figure 3H**, RNA pull-down depicted that biotin-labeled WT-LINC00494 probes pulled down greater amounts of NFκB1. Taken altogether, these results confirmed that LINC00494 promoted NFκB1 expression through binding to NFκB1 protein.

### Enrichment of NFκB1 at the FBXO32 Promoter Region Reduces Its Activity in Ovarian Cancer Cells

**Figure 4:**
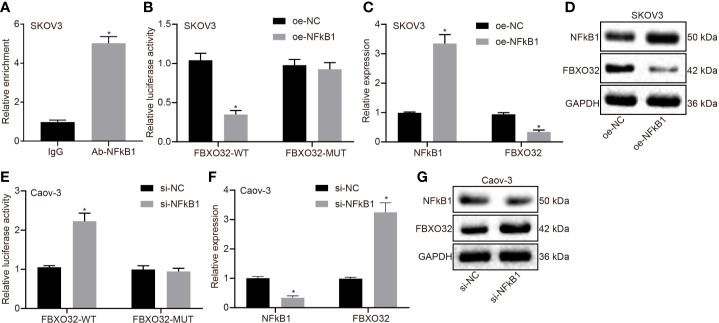
NFκB1 could bind to FBXO32 promoter and inhibit its expression. **(A)** SKOV3 cells were subject to ChIP assays using anti-NFκB1 or normal mouse IgG. We found anti-NFκB1 immunoprecipitated more FBXO32 relative to IgG. **(B)** The luciferase luminescence at the promoter of the reporter gene containing FBXO32-WT and FBXO32-MUT in oe-NFκB1-treated SKOV3 cells. **(C)** Real-time examined the expression of NFκB1 and FBXO32 in oe-NFκB1-treated SKOV3 cells. **(D)** Immunoblots of NFκB1 and FBXO32 in oe-NFκB1-treated SKOV3 cells. **(E)** The luciferase luminescence at the promoter of the reporter gene containing FBXO32-WT and FBXO32-MUT in si-NFκB1-treated Caov-3 cells. **(F)** Real-time examined the expression of NFκB1 and FBXO32 in oe-NFκB1-treated SKOV3 cells. **(G)** Immunoblots of NFκB1 and FBXO32 in oe-NFκB1-treated SKOV3 cells. **(A)** * indicates *p* < 0.05 compared with normal IgG; **(B, C)** * indicates *p* < 0.05 compared with empty vectors; **(E, F)** * indicates *p* < 0.05 compared with scramble siRNA. Unpaired t-test was used for statistical analysis.

Since we had demonstrated NFκB1 to be a transcriptional factor regulated by LINC00494, we next investigated whether FBXO32 is the downstream target gene of NFκB1. As demonstrated by ChIP assay, anti-NFκB1 immunoprecipitated more FBXO32 relative to IgG antibody in in SKOV3 cells (**Figure 4A**). The luciferase activity of the reporter containing FBXO32-WT decreased in SKOV3 cells following NFκB1 overexpression (**Figure 4B**). Real-time qPCR and immunoblotting analysis both demonstrated diminished expression of FBXO32 in SKOV3 cells following NFκB1 overexpression (**Figures 4C, D**). Likewise, we observed enhanced luciferase activity at the promoter of the reporter containing FBXO32-WT in NFκB1 knockdown Caov-3 cells (**Figure 4E**). Upon treatment with si-NFκB1 in Caov-3 cells, protein and mRNA expression of FBXO32 was elevated (**Figures 4F, G**). In conclusion, NFκB1 could bind to FBXO32 promoter and inhibit its expression.

### LINC00494 Inhibition of FBXO32 Facilitated Ovarian Cancer Cell Migration and Invasion

**Figure 5:**
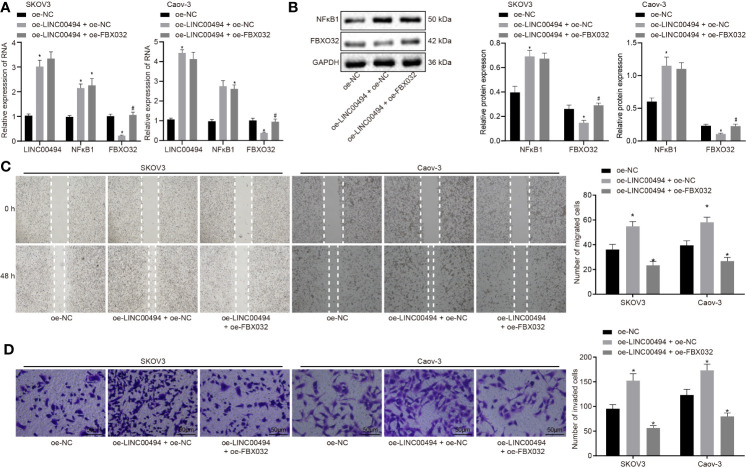
LINC00494 promotes migration and invasion of ovarian cancer cells SKOV3 and Caov-3 through NFκB1/FBXO32. **(A)** Real-time qPCR examined the expression of LINC00494, NFκB1 and FBXO32 in SKOV3 cells treated with vectors containing oe-LINC00494 and/or oe-FBXO32. **(B)** Expression of NFκB1 and FBXO32 proteins determined by immunoblotting analysis in SKOV3 cells treated with vectors containing oe-LINC00494 and/or oe-FBXO32. **(C)** The migration of SKOV3 cells treated with vectors containing oe-LINC00494 and/or oe-FBXO32 measured by scratch healing test. **(D)** Representative images (× 200) Transwell assay of SKOV3 cells treated with vectors containing oe-LINC00494 and/or oe-FBXO32 and corresponding quantification. * indicates *p* < 0.05 compared with empty vectors; # indicates *p* < 0.05 compared with expression vectors containing the LINC00494 alone. Tukey’s test-corrected one-way ANOVA was used for statistical analysis.

Our study aim was to study the effects of FBXO32 on ovarian cancer cells and investigate whether FBXO32 is responsible for the regulation of LINC00494 on ovarian cancer cells. We performed a rescue experiment in which SKOV3 and Caov-3 cells were transfected with vectors containing overexpression of LINC00494 and/or FBXO32. Real-time qPCR and immunoblotting analysis (**Figures 5A, B**) both demonstrated increased expression of LINC00494 and NFκB1 as well as decreased expression of FBXO32 upon transfection with oe-LINC00494. Combined treatment with oe-LINC00494 and oe-FBXO32 induced an increase of FBXO32 expression. Results from scratch and transwell invasion assays depicted that oe-LINC00494 treatment enhanced capability of migration and invasion, but the addition of oe-FBXO32 reversed the enhanced migration and invasion of cancer cells (**Figures 5C, D**). Thus, LINC00494 promoted SKOV3 and Caov-3 cell migration and invasion by negatively regulating FBXO32 *via* NFκB1 regulation.

### LINC00494 Facilitated *In Vivo* Tumorigenesis of SKOV3 Cells in Nude Mice

**Figure 6:**
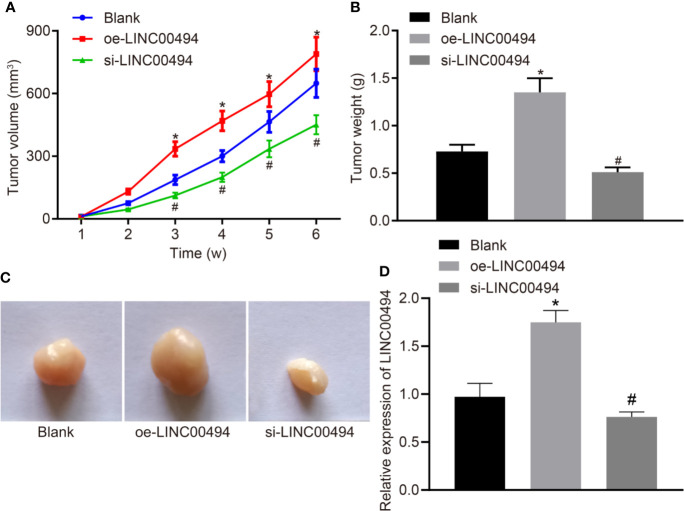
LINC00494 facilitates *in vivo* tumorigenesis of SKOV3 cells in nude mice. **(A)** The growth of subcutaneous xenotransplanted tumors of SKOV3 cells with stable oe-LINC00494 and si-LINC00494 in nude mice at indicated time points. **(B)** The weight of subcutaneous xenotransplanted tumors of SKOV3 cells with stable oe-LINC00494 and si-LINC00494 in nude mice. **(C)** The volume of subcutaneous xenotransplanted tumors of SKOV3 cells with stable oe-LINC00494 and si-LINC00494 in nude mice. **(D)** LINC00494 expression in tumor tissues upon treatment with oe-LINC00494 and si-LINC00494. * indicates *p* < 0.05 compared with empty vectors by unpaired t-test. N = 8, each group. * indicates p < 0.05 compared with the blank group; # indicates *p* < 0.05 compared with the oe-LINC00494 group.

Finally, we xenografted LINC00494-overexpressed and LINC00494 knockdown SKOV3 cells into nude mice to examine the effect of LINC00494 on the tumorigenesis of SKOV3 cells. We found that overexpression of LINC00494 increased the growth of subcutaneous xenotransplanted tumors of SKOV3 cells in the nude mice but si-LINC00494 slowed the growth of the mouse tumors compared to blank group (**Figure 6A**). Analysis at six weeks after implantation showed that overexpression of LINC00494 increased tumor weight and volume while silencing of LINC00494 had opposite effects (**Figures 6B, C**). Besides, real-time qPCR indicated higher expression of LINC00494 in tissues of mice treated with oe-LINC00494 and lower expression in mice treated with-si-LINC00494 (**Figure 6D**). Taken together, overexpression of LINC00494 promoted the xenograft tumor growth and silencing of LINC00494 exerted an opposite effect.

## Discussion

As revealed from *Global Cancer Statistics 2018*, there were 295,414 new cases of malignancies occurring in the ovary and 184,799 related deaths reported, accounting for 1.6% and 1.9% respectively of all cases the world over (15). Recent findings have emerged concerning the implications and potential therapeutic significance of multiple dysregulated lncRNAs in ovarian cancer (16, 17). Therefore, in the current investigation we endeavored to unravel the functional role of LINC00494 in ovarian cancer and to explore the underlying regulatory mechanisms. Collectively, the experimental data demonstrated that silencing LINC00494 had anticancer effects by upregulating FBXO32 *via* NFκB1.

We found in this study that LINC00494 was highly expressed in ovarian cancer tissues and cells. Similar to our results, LINC00152 has been indicated to be expressed at a high level in ovarian cancer in association with late cancer stage while silencing LINC00152 exerted inhibitory effects on cell proliferation, induced cell cycle arrest and encouraged rise to cell apoptosis (18, 19). However, that previous work had not documented whether cell migration and invasion were vulnerable to dysregulated LINC00152 expression. The further functional experiments in our study showed that knockdown of LINC00494 upon treatment with si-LINC00494 indeed suppressed malignant cell migration and invasion. Largely in agreement with our present results, downregulation of LINC01296 can abrogate the malignant cellular potential of migration and invasion in ovarian cancer (20). Similarly, LINC00319 has also been demonstrated to encourage proliferation, migration and invasion of ovarian cancer cells, thus exacerbating the progression and development of ovarian cancer (21). More importantly, inhibition on migration and invasion of ovarian cancer cells triggered by downregulated LINC00284 is reportedly dependent on the recruitment of NFκB1 and reduced expression of mesoderm-specific transcript (22). The lncRNAs are recognized to harbor actions as transcription promoters or enhancers of other RNAs encode proteins (23). Therefore, we performed subsequent experiments for exploring the underlying regulatory mechanism of aberrantly expressed LINC00494 in ovarian cancer.

Accordingly, further mechanistic investigations showed that LINC00494 recruited transcription factor NFκB1 to bind on the promoter region of FBXO32, which inhibited the transcription of FBXO32, while suppressing the progression and development of ovarian cancer. The NF-κB signaling pathway has been depicted to play an oncogenic role in ovarian cancer, in consideration of its demonstrated anti-apoptotic and immunomodulatory properties, thus presenting an attractive potential therapeutic target for treating ovarian cancer (24). A variety of lncRNAs serve as multipotent modulators of the NF-κB signaling pathway, and their interplay may be of therapeutic significance to oncogenesis (25). Likewise, the crosstalk between NF-κB and lncRNA HOX transcript antisense RNA has been clarified in a previous study that upregulated expression of NF-κB target genes contributes to cellular senescence in ovarian cancer (26). That finding is just in line with present results that NFκB1 was highly expressed in ovarian cancer and positively regulated by LINC00494 as its target gene. In addition, FBXO32 targets IκBα proteasome degradation in breast and human kidney transformed cells, while FBXO32 contributes the activation of NF-κB pathway in various tissues, while also exacerbating inflammation and genotoxic stress in breast cancer MCF7 cells (27). FBXO32 is activated under various stresses, making it a general marker of cellular stress responses. It is noted that FBXO32 is a specific stress response gene that activates the protective NF-κB pathway of breast cancer cells upon challenge with genotoxic and inflammatory stress (27). Moreover, recent research identifies FBXO32 as a functional tumor suppressor, whereby re-expression of epigenetically silenced FBXO32 in ovarian cancer cells facilitates cell sensitivity to cisplatin (12). In this study, we found that LINC00494 can bind to the transcription factor NFκB1, and that NFκB1 then binds to the FBXO32 promoter, aggravating ovarian cancer. Based on previous studies and present findings, we speculate that stressed ovarian cells may exploit this axis to resist ontogenesis.

## Conclusion

To conclude, the above-mentioned findings elaborated the anti-cancer action of downregulation of LINC00494 in ovarian cancer by curbing migration and invasion of cancer cells, while NFκB1-mediated overexpression of FBXO32 had the capability to override the favorable effects induced by LINC00494 knockdown. Notably, the identification of LINC00494 as a new regulator of ovarian cancer progression offers a fresh molecular insight, with some potential for translational for ovarian cancer. However, due to the limited time and funding, we are not allowed to explore the respective roles of FBXO32 and NFκB1 in ovarian cancer, which will be further studied in our future research for better clinical application of LINC00494 knockdown.

## Data Availability Statement

The raw data supporting the conclusions of this article will be made available by the authors, without undue reservation, to any qualified researcher.

## Ethics Statement

Animal studies were performed with the approval from the Institutional Animal Care and Use Committee of the First Hospital of Jilin University.

## Author Contributions

YHS and YS designed the study. HZ and JL collated the data, carried out data analyses, and produced the initial draft of the manuscript. YHS and YS contributed to drafting the manuscript. All authors contributed to the article and approved the submitted version.

## Conflict of Interest

The authors declare that the research was conducted in the absence of any commercial or financial relationships that could be construed as a potential conflict of interest.
